# Epidural haemorrhage during embolisation: a rare complication of intra-arterial embolisation of vertebral metastases

**DOI:** 10.2349/biij.7.4.e26

**Published:** 2011-10-01

**Authors:** H Hashim, KA Abdul Kadir

**Affiliations:** 1 Faculty of Medicine, Universiti Teknologi MARA, Selangor, Malaysia; 2 Department of Biomedical Imaging, Faculty of Medicine, University of Malaya, Kuala Lumpur, Malaysia

**Keywords:** embolisation, metastasis, complications

## Abstract

Pre-operative embolisation of vertebral metastases has been known to effectively devascularise hypervascular vertebral tumours and to reduce intra-operative bleeding. However, the complications that occur during the procedure are rarely reported. This case study attempts to highlight one rare complication, which is epidural tumoural haemorrhage intra-procedure. It may occur due to the fragility of the tumour and presence of neovascularisation. A small arterial dissection may also have occurred due to a slightly higher pressure exerted during injection of embolising agent. Haemostasis was secured via injection of Histoacryl into the area of haemorrhage. The patient was able to undergo the decompression surgery and suffered no direct complication from the haemorrhage.

## CASE REPORT

Mrs MA is a 50-year-old female who was diagnosed with right breast carcinoma with metastases in the thoracic spine. Following decompression and internal fixation at T10-T12 and L3-L5 vertebral bodies, her lower limb weakness improved and she was able to achieve independence in her daily living activities. However, she presented again with a one-week history of bilateral lower limb weakness, which was progressively worsening.

During admission, an MRI of the thoracolumbar spine showed metastatic deposits in T9, T10, T12, L1 to L3 and L5 vertebral bodies. There was also T9 compression fracture causing marked narrowing of the spinal canal (AP diameter 0.4 cm) and cord oedema.

**Figure 1:**
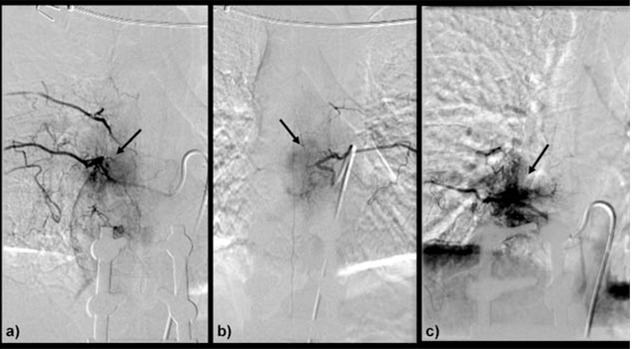
Pre-embolisation angiogram shows multiple tumour blushes (arrows) from: a) right T8, b) left T8 and c) right T10 intercostal arteries.

**Figure 2:**
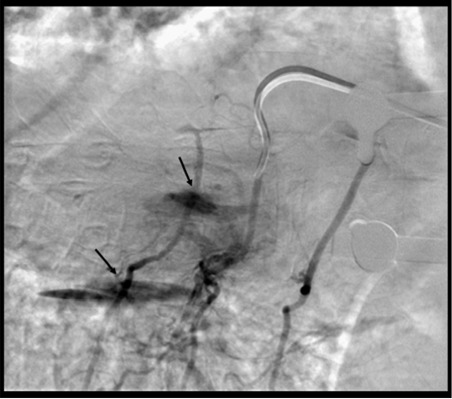
Tumour bleed (arrow) during embolisation of right T8 intercostal artery.

**Figure 3:**
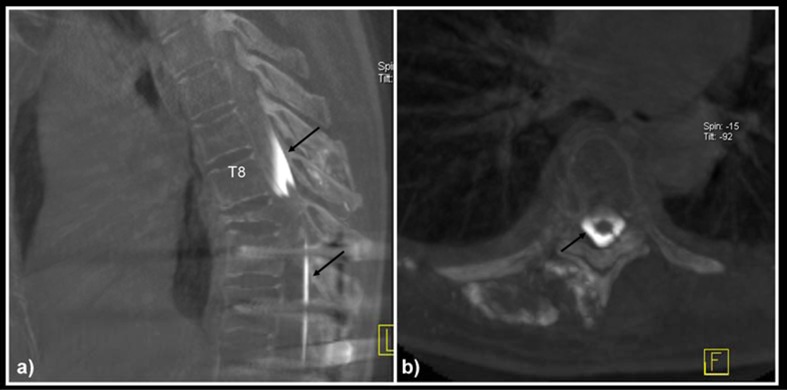
Dyna-CT on: a) sagittal and b) axial view confirmed tumoural bleed which extends into the epidural and intrathecal space (arrows).

She was scheduled for emergency decompression of the T9 vertebral body. A pre-operative intra-arterial embolisation of the tumour was requested to aid in the surgery. The C-arm system used was a single-plane AXIOM Artis *d*Fa C-Arm Angiography System (Siemens, Germany). Image acquisition was done using a dynamic flat panel detector system with 48cm diagonal entrance plane producing an image of 1920 × 2480 matrix with 154 μm pixel size. Using a 5F Shepherd Hook catheter, angiogram was performed and showed multiple tumour blushes from T8–T10 intercostal arteries (Figure 1). The anterior spinal artery was seen arising from the left T8 intercostal artery. With a 2.7F Progreat microcatheter, a selective catheterisation and embolisation into the right T8 and T9 intercostal arteries was performed. However, during the embolisation of the right T8 artery, tumour bleed was noted (Figure 2). A mixture of 0.5ml Histoacryl and 1ml Lipiodol has been prepared in advance, producing a concentration of 25% Histoacryl. Small amounts of this premixed Histoacryl was injected into the right T8 intercostal artery slowly until haemostasis was secured. Dyna-CT (on the same system using 5 seconds-1k DS protocol) confirmed tumoural bleed which extends into the epidural and intrathecal space with contrast extending to T7 level (Figure 3). Contrast extravasation was also noted to the right erector spinae muscle at T8 level.

There was no deterioration of the neurological status and vital signs remained stable throughout procedure. She underwent the decompression surgery without any further complications. During her subsequent follow-up, she was able to mobilise herself with a walking aid.

## DISCUSSION

The skeletal system is the third most common site of cancer metastases, after the lungs and the liver [1]. 80% of skeletal metastases arises from breast, lung, prostate and renal cells [2]. In 1940, Batson postulated that venous spread is a potential pathway for spinal metastases. The presence of a valveless epidural venous plexus (Batson’s plexus) allows diversion of blood into the plexus and provides a potential pathway for metastatic deposits [2].

Vertebral metastases can be treated for palliation or local tumour control. Pre-operative embolisation facilitates surgical resection by reducing intra-operative bleeding, ensuring a better view of the tumour during surgery and reducing the tumour size, thus making total resection possible [1, 3, 4].The most frequently used embolic agent reported is PVA particles, likely due to its ease of delivery and ability to achieve distal embolisation with a favourable safety profile [1, 3, 5, 6].

Identification of the anterior spinal arteries is important as incidental embolisation of one of these arteries may injure the pyramidal tracts and cause severe motor deficit. The presence of an anterior spinal artery at the same arterial pedicle as the feeding artery to the tumour is a contraindication for embolisation [1].

The indication for pre-operative spinal embolisation is limited, thus there are not many articles regarding its complications. Most of the literature discussed its influence on peri-operative haemorrhage and reported that it is deemed to be safe and effective. Prabhu *et al*. performed the procedure on 51 patients. Two patients demonstrated small asymptomatic lacunar infarcts on MR imaging but, otherwise, no major complications occurred as a result of embolisation [4]. Hai Bin Shi *et al*. reviewed 18 cases and reported no complications within 24 hours after embolisation [1]. The few reported complications in the literature include dermatomal paraesthesia, transient myelopathy resulting from tumour swelling which was relieved by surgery, and one case of permanent paraparesis [1]. Finstein *et al*. reported a case of post-embolisation paralysis in a patient with thoracolumbar giant cell tumour. At 6 months follow -up, the patient was disease-free but without neurological function from T12 and below [7].

One possible explanation for the spontaneous haemorrhage that occurred during this embolisation is the fragility of the tumour and presence of neovascularisation resulting in multiple fragile vessels. Following implantation of metastatic cells, a tumour has to obtain its own vascular supply for it to become clinically significant. Secretion of a tumour angiogenesis factor attracts vessels to the small tumour colony and generates formation of numerous new but fragile vessels [2].

The rate and pressure of injection via the catheter may also contribute towards the haemorrhage. It is possible that, during the procedure, a slightly higher pressure is exerted during injection of PVA articles, resulting in small arterial dissection.

The immediate availability of glue (e.g.: Histoacryl) to aid in haemostasis is stressed in this case report. With the catheter already in place, the radiologist was able to secure haemostasis the moment he noted the haemorrhage by injecting Histoacryl via the catheter. This is important, not just in embolisation of spinal metastases, but also in other transarterial embolisation where arterial dissection or haemorrhage is a possible complication.

Percutaneous or direct embolisation of vertebral tumours is another alternative to reduce the vascularity of vertebral tumours. In cases where the tumour feeders are atherosclerotic, tortuous, anatomically difficult to be super-selectively catheterised or where adequate transarterial embolisation is only possible at the risk of compromising vital spinal cord feeders, this method is useful [8]. Various embolic agents have been used, such as PVA, n-butyl cyanoacrylate (NBCA), coils and Gelfoam [8–10]. Schirmer *et al*. described a method of combining transarterial embolisation with direct percutaneous vertebral NBCA embolisation, which achieved a closer to true end-organ embolisation rather than the more proximal occlusion usually achieved by transarterial embolisation [10].

Despite having a small epidural bleed following this complication, the patient was able to undergo the decompression surgery. With physiotherapy, she was able to mobilise herself with a walking aid. Pre-operative embolisation is still a safe and effective procedure for tumour devascularisation and thus aids in surgery. Physicians and radiologists should be aware of this rare complication.
